# Effect of different cavity liners on nanocomposite resin shear bond strength to dentine

**DOI:** 10.6026/973206300200297

**Published:** 2024-03-31

**Authors:** Mounika Veeraiyan, Divakar KP, Sarang Sharma, Sharan Priya, Shruti A Nayak, Kanika Singh Dhull, Pratik Surana

**Affiliations:** 1 Department of Conservative Dentistry & endodontics, ESIC Dental College & Hospital, Kalaburagi, Karnataka, 585106, India; 2 Department of Conservative Dentistry & Endodontics, ESIC Dental College & Hospital, Rohini, Delhi, 110089, India; 3 Department of Conservative Dentistry, Endodontics, All India Institute of Dental Sciences, Bibinagar, Hyderabad, 508126, India; 4 Department of Pedodontics and Preventive Dentistry, Kalinga Institute of Dental Sciences, KIIT Deemed to be University, Bhubaneswar, India; 5 Senior Lecturer, Department of Pedodontics and Preventive Dentistry, Maitri College of Dentistry and Research Centre, Durg, Chhattisgarh, India

**Keywords:** Apacal ART, Biodentin, Giomer, Cavity liner, Shear bond strength

## Abstract

The effect of different cavity liners on the shear bond strength of nanocomposite to dentin is of interest. A total of sixty extracted caries-free maxillary, mandibular molars were randomly assigned to four groups in the following manner Group 1: control (no cavity liner), group 2: Biodentin, group 3: Apacal ART and Group 4: Giomer. Following the application of different cavity liners based on the groups, restoration was carried out using nanocomposite resin using the total-etch Tetric N bond adhesive. The samples were thereafter subjected to a shear bond strength test at a cross-head speed of 1 mm/min until bond failure occurred, utilizing the universal testing machine. The one-way ANOVA test and the post hoc test were used to evaluate the data for pairwise group comparisons. Compared to the control group, all groups showed lower shear bond strength to dentin, irrespective of the type of liner. Apacal ART showed higher shear bond strength followed by giomer and biodentin. However, there's no apparent statistical difference between the groups.

## Background:

Protective cavity liners are placed inside deep cavities to protect the pulp from various stimuli and promote the development of reparative dentine. By sealing dentinal tubules, these materials protect the pulp from irritants, microorganisms, and thermo-mechanical stimuli. [1] Traditionally, calcium hydroxide was used as a pulp-capping agent since it is antimicrobial, has an alkaline pH, and stimulates mineralization. Nevertheless, calcium hydroxide's work as a pulp-capping agent has decreased as a result of the tunnel defects and micro-leakage that have been recorded. Several materials have been used as cavity liners; these materials include Theracal LC, Apacal LC, Biodentine, MTA, RMGIC, and giomer, as well as direct and indirect pulp capping [1,2]. Biodentine (Septodont, France) was introduced in 2011. It is cement made of tricalcium silicate. It can produce mineralized tissue, preserve pulp vitality, and odontoblast layer integrity, and enhance mechanical properties. It is therefore recommended as a pulp capping agent [3, 4]. Giomers combined the characteristics of composite resin with glass ionomer cement. It is composed of a bis-GMA matrix with bioactive glass fillers and fluoride. Light-activated cement. [5] ApaCal ART (A.ART) is a pulp capping material with light-cured resin-modified tricalcium phosphate and added hydroxyapatite. T is claimed to offer benefits such as rapid dentin bridge development and calcium ion release. It can be used as a direct and indirect pulp capping agent for deep cavities while cavity liners are not always required with composite resin restorations; they are in certain clinical situations. [6] There is a lack of knowledge on the behavior of lining materials beneath composite restorations. Therefore, it is of interest to evaluate the effects of different liners on the shear bond strength of the liner composite to dentin. The null hypothesis is that there is no difference between the shear bond strength of different cavity liners to composite resin.

## Materials and methodology:

The sample size calculation was done in G*power version 3.0. The sample size was calculated, keeping the effect size as 0.6 with an alpha error of 0.05 and power of 0.95, the total sample size was calculated as 60 which is divided as 15 in each group. In this in vitro experimental study, sixty caries-free human maxillary and mandibular molar teeth were extracted for orthodontic purposes or periodontal disease. After cleaning, teeth were stored for two weeks at 37°C in distilled water. Using a circular polishing machine and 180-grit sandpaper, the occlusal surface of the teeth was completely removed from their enamel, revealing 7 mm of smooth dentin. The process was done under water cooling. Then, brass molds measuring 2.5 x 3.5 centimeters were filled with self-curing acrylic resin and molars were inserted. To normalize the smear layer under water lubrication, a polishing machine equipped with 600-grit sandpaper was utilized.

Specimens were randomly divided into 4 based on the groups; the samples were kept in distilled water at 37°C. The area and volume of composite restorative materials (4 mm height and 4 mm internal diameter) and liners (1.5 mm height and 1.5 mm internal diameter) were standardized using the polyethylene tube. Following the application of the appropriate lining materials (group 1- no liner, group 2- Biodentin, group 3- Apacal ART, and group 4 - Giomer), 37% phosphoric acid gel was used to etch the dentin surfaces and the liners in each study group for 15 seconds and rinsed with water. A bristle brush was used to apply two coats of total-etch Tetric N bond over the surrounding dentin surface and liner. It underwent a 15-second rub, a 5-second mild air drying period, and a 10-second light-curing session using a light source with an 800 mW/cm2 light intensity at a 1-mm standard distance for 40 seconds.

Following the incremental method, the polyethylene tube (4 mm in height and 4 mm in internal diameter) was positioned over the lining material and filled with composite resin. Each increment was then cured for 40 seconds using a light curing unit. The samples were subsequently subjected to thermo cycling, which involved 5000 cycles at 5 to 55°C with a 30-second remain and transfer period. A universal testing apparatus utilizing a 50 kg load cell was utilized to evaluate the shear bond strength. The crosshead speed was set at 1 mm/min until the bond started to fail.

## Results:

Descriptive statistics was expressed using mean and standard deviation. Inferential statistics was done by using a one-way ANOVA test followed by a post hoc test to assess the comparison between the groups. To analyze the data SPSS (IBM SPSS Statistics for Windows, Version 26.0, Armonk, NY: IBM Corp. Released 2019) is used. The significance level is fixed at 5% (α = 0.05). P-value <0.05 is considered to be statistically significant.

## Discussion:

The success of restorations and the preservation of pulp vitality depend on the liners' bond strength to dentine and restorative materials, as well as their solubility during the etching process. This is because the pulp capping materials may have an impact on the longevity and state of the tooth-restoration interface [7]. According to the study's findings, group 1 (control) had a better bond strength mean value of 2.9071 (no pulp liner), which was followed by group 3 (Apacal ART) with a mean value of 2.5214, group 4 (Giomer) with a mean value of 2.1179, and group 2 (Biodentin) with a mean value of 2.05. Shear bond strength was higher in Group 3 Apacal LC than in the other groups (Table 1). Apacal LC may have a greater affinity with dentin since it contains hydroxyl apatite and calcium phosphate.[6] Groups 3 (Apacal ART), 4 (Giomer), and 1 (control group) were compared pairwise, and the results indicated that there is no statistically significant difference between the groups. Giomer (Group 4) showed less shear bond strength compared to Group 2 (Apacal ART) and Group 1 (control), and in pair-wise comparison, there is no difference between the groups typically, giomer binds to dentin via dentin adhesive-mediated micromechanical connections to collagen fibrils. The fluoride level of the giomer may exacerbate micro leakage and have a detrimental effect on the strength of the bond between the giomer and tooth structure [7, 8, 9]. Comparing Group 2 (Biodentine) to the other groups, the bond strength values were noticeably lower. Following the manufacturer's specified 12-minute setting period, the composite was applied over Biodentine in our investigation. Previous research indicates that the bond strength between restorative materials and biodentine may be impacted by the biodentine's setting reaction. Moreover, the precise process by which Biodentine adheres to dentine remains unclear. It has been suggested that a combination of the chemical and micromechanical bonding that cement tags inserted into the dentinal tubules offer is what causes this bonding [10,11, 12,13]. Lower SBS values may have occurred in this study if the composite had been placed before the mechanical bond between the biodentine and dentin had fully formed and matured, which happened after 12 minutes. Between the groups, there is no statistically significant difference in a pairwise comparison (Table 2). It could be because of the limited sample size. Future research must focus on a larger number of sample sizes. In contrast to alternative testing techniques, the macro-shear test was employed in our investigation because of its benefits, which include minimal force application changes and simple setup and sample preparation for shear tests.

## Conclusion:

Data shows that the use of various types of liners can have distinct effects on the bond strength of composite resin to dentin in comparison to their not being used. When compared to other groups, Apacal ART demonstrated the strongest shear bond strength for composite resin.

## Figures and Tables

**Table 1:** Comparison between the study groups

**VARIABLES**	**GROUP 1 (CONTROL)**	**GROUP 2 (BIODENTIN)**	**GROUP 3 (APACAL ART)**	**GROUP 4 (GIOMER)**
Mean	2.9071	2.05	2.5214	2.1179
Std. Error of Mean	1.48429	0.40154	0.21308	0.19665
Std. Deviation	5.55372	1.50243	0.79728	0.73579
Variance	30.844	2.257	0.636	0.541
Range	21.75	3.75	3	3.05
Minimum	0.25	0.25	1	0.75
Maximum	22	4	4	3.8
F-value	0.25			
P-value	0.86			

**Table 2:** Pairwise comparison between the study groups

**SURFACE**	**GROUPS**	**Mean Difference (I-J)**	**Std. Error**	**P-value**	**95% Confidence Interval**	
					**Lower Bound**	**Upper Bound**
Apacal LC	Giomer	0.40357	1.10644	0.983	-2.533	3.3402
	Control	-0.38571	1.10644	0.985	-3.3223	2.5509
	Biodentin	0.47143	1.10644	0.974	-2.4652	3.408
Giomer	Control	-0.78929	1.10644	0.891	-3.7259	2.1473
	Biodentin	0.06786	1.10644	1	-2.8688	3.0045
Control	Biodentin	-0.85714	1.10644	0.866	-3.7938	2.0795
